# Entrapment as a mediator of suicide crises

**DOI:** 10.1186/s12888-018-1587-0

**Published:** 2018-01-08

**Authors:** Shuang Li, Zimri S. Yaseen, Hae-Joon Kim, Jessica Briggs, Molly Duffy, Anna Frechette-Hagan, Lisa J. Cohen, Igor I. Galynker

**Affiliations:** Department of Psychiatry, Beth Israel Medical Center NY, 1st Avenue and 15th Street, New York, NY 10003 USA

**Keywords:** Suicide, Suicidal ideation, Entrapment, Emotional pain, Suicide crisis

## Abstract

**Background:**

Prior research has validated the construct of a suicide crisis syndrome (SCS), a specific psychological state that precedes and may precipitate suicidal behavior. The feeling of entrapment is a central concept of the SCS as well as of several other recent models of suicide. However, its exact relationship with suicidality is not fully understood. In efforts to clarify the exact role of entrapment in the suicidal process, we have examined if entrapment mediates the relationship of other components of the SCS, including ruminative flooding, panic-dissociation, fear of dying and emotional pain, with suicidal ideation (SI) in recently hospitalized psychiatric inpatients.

**Methods:**

The Suicide Crisis Inventory (SCI) and Beck Scale for Suicidal Ideation (BSS) were administered to 200 high-risk adult psychiatric inpatients hospitalized following SI or suicide attempt, assessing SCS and SI levels at admission, respectively. The possible mediation effects of entrapment on the relationship between the other components of the SCS and SI at admission were evaluated.

**Results:**

Entrapment significantly and fully mediated the relationship of ruminative flooding, panic-dissociation, and fear of dying with SI, with no direct relationships between these variables and SI reaching statistical significance. Further, no reverse mediation relationships between these variables and SI were found, indicating that the mediation effects of entrapment were unidirectional. While entrapment did mediate the association between emotional pain and SI, the direct relationship between emotional pain and SI was also significant. Moreover, in reverse mediational analysis, emotional pain was a partial mediator of the relationship between entrapment and SI.

**Conclusion:**

Entrapment and emotional pain may have a more direct association with SI than the other components of the SCS, including ruminative flooding, panic-dissociation, and fear of dying, the effects of which are mediated by the former. This suggests entrapment and emotional pain may represent key symptomatic targets for intervention in acutely suicidal individuals. Further research is needed to determine the relationship of these constructs to suicidal behavior.

**Electronic supplementary material:**

The online version of this article (10.1186/s12888-018-1587-0) contains supplementary material, which is available to authorized users.

## Background

Suicide is a major public health concern, ranking as the 10th leading cause of death in the United States, and accounts for over 42,773 deaths in the US each year [1]. Suicide is a complex behavior reflecting a multi-level interplay of biological, psychological, social, and material factors such as access to means. Although the predictive value of long-term risk factors including biomarkers (e.g., serotonin levels), demographic factors (sex, age), and clinical factors (diagnosis of mental illness, prior suicide attempt) has been well established [2, 3], a recent meta-analysis of 50 years of research on risk factors for suicidal thoughts and behaviors (STB) highlights the consistently small effect sizes of such risk factors and that no risk factor category or subcategory is substantially stronger than any other [4]. Likewise, reliable predictors of acute suicide risk have also remained elusive. Recently, however, a small but growing body of evidence suggests the existence of an acute suicide crisis syndrome (SCS) [5, 6], characterized by the experience of entrapment accompanied by intense negative affect, loss of cognitive control, hyper-arousal, and social withdrawal, which may precipitate the transition from chronic suicidal ideation (SI) to acute suicide attempt (SA) [7–12]. Our previous work on a series of instruments aiming to define the SCS has shown several clinical factors, proposed as criterion symptoms of a clinical syndrome that characterizes the suicidal crisis, are indeed related to each other and short-term risk for suicidal behavior. These include the constructs of entrapment, ruminative flooding (a cognitive control symptom), panic-dissociation and fear of dying (hyper-arousal symptoms), and emotional pain (an intense negative affect symptom) [10–13] and encompass the affective, cognitive, and somatic aspects of the hypothesized SCS. Notably, the Suicide Crisis Inventory (SCI) has demonstrated strong internal consistency, and significant predictive as well as incremental predictive validity over standard suicide predictors (SI, depression, state and trait anxiety) for short-term post-discharge suicidal behavior [12].

The first construct, entrapment, has recently been proposed as a key psychological element of several models of suicidal behavior, notably the Arrested Flight/Cry of Pain model by Williams and Pollock [14] and O’Connor’s Integrated Motivational-Volitional Theory (IMV) [15]. According to these models, entrapment, defined as a felt urgency to escape from an unbearable situation from which there is no perceived escape [16], could be a core psychological mechanism in causal pathways to suicide. In agreement with this hypothesis, a strong positive association was found between perceptions of entrapment and suicidality in a range of subacute outpatient populations [17–23]. O’Connor’s group also demonstrated that entrapment added incremental predictive validity for suicidal behaviors over depression, hopelessness, SI, and the frequency of previous suicide attempts [24]. Similar to their findings, in our group’s latest study of the SCS, entrapment was the strongest individual predictor of suicidal behavior within the 2 months following discharge from an inpatient unit [12].

Surprisingly, although entrapment is a core concept of many models of suicide and of the hypothesized SCS, its role as a possible mediator of the relationship between other acute risk factors and SI/suicidal behavior has never been examined in *acutely suicidal patients.* The IMV model [15] posits that ruminations increase the likelihood of stressful experiences leading to perceptions of entrapment, which in turn may trigger SI. Alternatively, entrapment may lead to a cognitive search for escape routes resulting in rumination, thus triggering SI. Uncovering these relationships may not only help clarify theoretical models of suicidal behavior, but also establish important targets for therapeutic interventions in acutely suicidal individuals.

The second hypothesized component of the SCS, ruminative flooding, is characterized by uncontrollable perseverative thinking involving continual thoughts about the causes, meaning, and consequences of one’s negative mood. Ruminative flooding differs from simple ruminations in that it is experienced as an uncontrollable and overwhelming profusion of negative thoughts, often associated with somatic symptoms inside the head, such as headaches or head pressure [10]. Similar constructs have been associated with elevated suicide risk by other research teams [25, 26]. The third construct, panic-dissociation, describes somatic symptoms commonly associated with a panic-like dissociative state, and involves somatic experiences of unfamiliar sensations felt all over the body, and derealization [11, 13]. Similar constructs have also been described by other suicide researchers [27, 28]. The fourth construct, fear of dying, describes morbid cognitions during panic, which may mediate the transition from latent SI to active SI and suicide attempt in some depressed subjects [29–34].

Finally, the fifth hypothesized component of the SCS is emotional pain, a mixture of intense and painfully felt negative emotions such as guilt, shame, hopelessness, disgrace, rage, and defeat, which arises when the essential needs to love, to have control, to protect one’s self image, to avoid shame, guilt, and humiliation, or to feel secure are frustrated [35]. Emotional pain is similar to entrapment in that it is strongly correlated with but distinct from anxiety and depression, and can be so intense that the individual seeks to escape by – suicide [35–39]. However, those in emotional pain may lack the desperation caused by the perception that all escape routes are blocked, whereas this desperation and subsequent escape motivation are intrinsic components of entrapment.

Severe or pervasive SI is consistently found to be a significant predictor of subsequent suicidal behaviors [40]. Even passive ideation, such as a wish to die, has been identified as a risk factor for death by suicide [41]. Although the relationship between SI, suicidal behavior, and suicide is not straightforward, and several other symptom dimensions may synergistically influence suicide risk [20, 42], SI has a much higher base rate than suicides or suicide attempts and thus represents a scientifically useful and clinically meaningful modifiable element of suicide risk.

Given the evidence of the central role of entrapment in the suicidal process, the goal of the present report was to establish whether entrapment mediates the relationship between the other clinical factors of the SCS and SI in psychiatric inpatients recently hospitalized for suicide risk. As, the SCS is conceptualized as a clinical syndrome that activates SI, motivating the transition from SI to SA, in the context of the current study, severity of SI serves as a proxy for nearness to suicidal action. We therefore reanalyze the data from our published validation study of the SCI [12] to examine the interrelationships among the SCI subscales and severity of suicidal ideation. We hypothesized that entrapment will mediate the relationships between SI and ruminative flooding, panic-dissociation, fear of dying, and emotional pain.

## Methods

### Study subjects

Participants between the ages of 18–65 hospitalized for SI or SA were recruited from the inpatient psychiatric units at Mount Sinai Beth Israel (MSBI) in New York City between April 2013 and July 2015. Potential participants were identified and referred to the study by a clinician. Within 72 h of observation and admission to the psychiatric inpatient unit, a trained research assistant approached each eligible patient to explain the study, its aims, and the risks and benefits of participation. To avoid reporting bias that might be associated with presenting the study as explicitly focused on suicidality, the study was titled “Predicting Emotional Dysregulation: Internal Consistency and Predictive Validity of the Emotional Dysregulation Scale” and the phrases “SI” and “suicide attempt” were replaced with the term “emotional dysregulation” in the consent form. Following an explanation of what the study entailed, he or she was then presented with a consent form to review and sign, and study measures were completed concurrently.

Participants were excluded if they had cognitive or linguistic limitations precluding their understanding the consent or research questions, significant medical or neurological disease, or possible delirium that might interfere with study participation or informed consent. The study was approved by the MSBI Institutional Review Board, project #223–14.

### Measures

#### The suicide crisis inventory (SCI)

The 49-item SCI, comprises 5 subscales: entrapment (13 items), panic-dissociation (9 items), ruminative flooding (7 items), emotional pain (4 items), and fear of dying (3 items). The remaining items contribute to the total SCI score but are not assigned to a subscale. Items are rated by self-report on a five-point scale ranging from ‘not at all = 0’ to ‘extremely = 4’. Each subscale, as previously reported, demonstrates good to excellent internal consistency in the study sample (entrapment: α = 0.946; panic-dissociation: α = 0.882; ruminative flooding: α = 0.892; emotional pain: α = 0.878 and fear of dying: α = 0.796) [12].

#### Suicidal ideation (SI)

Severity of SI was assessed with the Part 1 total score of the Beck Scale for Suicidal Ideation (BSS) [43], a 21-item self-report measure of SI. Scores on each item range from 0 to 2, with higher scores indicating greater severity of SI. Part 1 of the BSS was used because Part 2 is completed only if items 4 and 5 in Part 1 are scored >0, and thus total scores were not calculated for the entire sample.

#### Depression severity

Severity of depression (exclusive of suicidal ideation) at admission was assessed with the Beck Depression Inventory II (BDI) [44, 45], a 21-item self-report measure of depression with item 9 (suicidal ideation) excluded. Scores on each item range from 0 to 3, with higher scores indicating greater severity of SI.

### Statistical analysis

To investigate the hypothesis that the relationship between each construct and SI was fully mediated by the perception of entrapment, a simple mediation model was applied, with severity of SI as the dependent variable.

Direct, indirect and total effects together with their bias-corrected 95% confidence intervals were calculated based on 5000 bootstraps. The mediation effect was assessed by (a) Preacher & Kelley’s [46] Kappa-squared test, where κ^2^ = the proportion (ranging from 0 to 1) of the maximum possible indirect effect observed and (b) the completely standardized effect size (CS) interpreted relative to zero with larger values indicating larger effect size. In those tests, a bootstrapped 95% confidence interval (CI) excluding zero indicates statistical significance. The Sobel test was chosen as a conservative confirmation of significance of the mediation effect, with a threshold alpha value of 0.05 for significance. To test the unidirectionality of the mediation effects, ‘reversed’ mediation models were calculated with entrapment as the independent variable and each of the other SCS constructs as the mediator. If no mediation is found in these reversed models, a unidirectional mediation effect of entrapment is supported. In other words, if a mediation effect is significant but the reversed mediation is not, we can say with more confidence that (for example) entrapment accounts for the effect of ruminative flooding on SI and not the other way around.

Finally, in supplementary analyses we examined whether a) SI severity as assessed by the BSS and mediation effects differed between patients admitted in the context of recent suicide attempt and patients admitted with SI only, and b) mediation effects were retained when age and concurrent depression severity were controlled for.

All statistical tests were performed using the Statistical Package for the Social Sciences (SPSS), version 21. Mediation analyses were performed using Hayes [47] PROCESS module model 4 for SPSS.

## Results

### Demographics

Two hundred and one patients met inclusion criteria and provided informed consent. Data from one participant were excluded because the patient did not complete the BSS. Among these, 142 were hospitalized after presenting with SI, while 58 made attempts leading to their hospitalization. Table 1, summarized below, presents the demographic characteristics of the study participants as previously described [12]. The remaining sample of 200 participants was roughly 50% female and had a racial composition approximately equivalent to that of the general New York City population. Among the participants, nearly half had a unipolar mood disorder, and approximately one quarter had a psychotic or bipolar mood disorder, while roughly a third were diagnosed with other disorders such as an unspecified adjustment disorder. Over half also were diagnosed with a comorbid substance use disorder.

**Table 1:** Demographic characteristic of study participants

Age (years)	Mean = 35.3	SD = 13.4
Education (years)	Mean = 13.7	SD = 2.9
Variable	N	Percent
Gender		
Male	91	46.3
Transgender	2	1.0
Female	107	53.7
Race		
Asian	20	10.0
Black	43	21.5
White	76	38.0
Other	61	30.5
Ethnicity (Latino)	68	34.0
Marital status		
Never married	136	68.0
Married	25	12.5
Divorced/widowed/separated	39	19.5
Diagnosis		
Psychotic spectrum disorders	20	10
Bipolar spectrum disorders	26	13
Unipolar depressive disorders	84	42
Other disorders^a^	70	35
Substance use		
EtOH use disorder	75	37.5
Illicit substance use disorder	97	48.5
Any substance use disorder	120	60

^a^Mood disorder not otherwise specified, adjustment disorder, substance-induced disorders, personality disorder only, anxiety disorders

### Inter-correlations among each component of the suicidal crisis syndrome

Current SI and each of the subscales of the SCI had significant positive correlations with each other with r values ranging from 0.15 to 0.71. See Table 2.

**Table 2:** Inter-correlations among study variables (*N* = 200)

	Entrapment	Panic /Dissociation	Ruminative Flooding	Fear of dying	Emotional Pain	SI
Entrapment	1					
Panic/Dissociation	.510^***^	1				
Ruminative Flooding	.671^***^	.609^***^	1			
Fear of dying	.519^***^	.572^***^	.496^***^	1		
Emotional Pain	.708^***^	.500^***^	.619^***^	.454^***^	1	
SI	.422^***^	.228^**^	.316^***^	.145^*^	.417^***^	1

*SI* suicidal ideation

^*^p < 0.05 (2-tailed), ^**^p < 0.01 (2-tailed), ^***^p < 0.001 (2-tailed)

### Mediation analyses

#### Ruminative flooding

As indicated in Tables 3 and 4, the total effect of ruminative flooding on SI was significant at *p* < 0.001 with a standardized regression coefficient of 0.120. The direct effect of ruminative flooding on SI was not significant (95% CI including 0). In contrast, the indirect effect of ruminative flooding on SI was significant (95% CI did not include 0 and Sobel test: z = 4.140, *p* < 0.001), suggesting that entrapment fully mediates the relationship between ruminative flooding and SI with small to mediate effect size (CS = 0.256, κ^2^ = 0.202). As indicated in Table 11, the reversed mediation analysis with entrapment as the independent variable and ruminative flooding as the mediator showed no significant indirect effect (Sobel test: *p* = 0.494). Both the direct effect and the total effect of entrapment on SI were significant. These results show that entrapment fully mediates the relationship between ruminative flooding and SI, and conversely, that ruminative flooding is not a mediator of the relationship between entrapment and SI.

**Table 3:** Regression coefficients of entrapment and ruminative flooding predicting suicidal ideation

Predicted variable								
M(entrapment)					Y (suicidal ideation)			
Predictor variable		Coeff.	SE	P		Coeff.	SE	P
*X (Ruminative Flooding)*	*a*	1.192	0.094	<0.001	*c’*	0.023	0.033	0.494
*M (Entrapment)*		–	–	–	*b*	0.082	0.019	<0.001
*Constant*	*i*_*1*_	13.187	1.684	<0.001	*i*_*2*_	0.387	0.504	0.443
R^2^ = 0.450					R^2^ = 0.180			
F(1,198) = 162.000, *p* = <0.001					F(2,197) = 21.649, *p* < 0.001			

“*a*” is the regression coefficient predicting entrapment by ruminative flooding

“*b*” is the coefficient predicting suicidal ideation by entrapment

“*c’*” is the coefficient predicting suicidal ideation from ruminative flooding independent of entrapment

**Table 4:** Total, direct and indirect effects and effects sizes for indirect effects of ruminative flooding on suicidal ideation mediated by entrapment

	Raw effect	SE	lower	upper	*p*	CS	SE	κ^2^	SE	Z Score
Indirect effect	0.097	0.023	0.054	0.142	<0.001	0.256	0.061	0.202	0.047	4.140
Direct effect	0.023	0.033	−0.043	0.088	0.494					
Total effect	0.120	0.026	0.070	0.171	<0.001					

*SE* standard error, *lower* lower bound of 95% confidence interval, *upper* upper bound of 95% confidence interval, *CS* completely standardized effect size, *κ*^*2*^ Kappa-squared test, Z score by Sobel test

#### Panic-dissociation

As shown in Tables 5 and 6, the total effect of panic-dissociation on SI was significant at *p* = 0.001 with a standardized regression coefficient of 0.077. The direct effect of panic-dissociation on SI was not significant (*p* = 0.821). In contrast, the indirect effect of panic-dissociation on SI was significant (95% CI did not include 0 and Sobel test: z = 4.573, *p* < 0.001). This suggests that the effect of panic-dissociation on SI was fully mediated by entrapment.

**Table 5:** Regression coefficients of entrapment and panic-dissociation predicting suicidal ideation

Predicted variable								
M (entrapment)					Y (suicidal ideation)			
Predictor variable		Coeff.	SE	P		Coeff.	SE	P
*X (Panic dissociation)*	*a*	0.810	0.097	<0.001	*c’*	0.006	0.026	0.821
*M (Entrapment)*		–	–	–	*b*	0.088	0.016	<0.001
*Constant*	*i*_*1*_	23.164	1.408	<0.001	*i*_*2*_	0.469	0.489	0.339
R^2^ = 0.260					R^2^ = 0.174			
F(1,198) = 69.432, *P* = <0.001					F(2,197) = 21.394, *P* < 0.001			

“*a*” is the regression coefficient predicting entrapment by panic-dissociation

“*b*” is the coefficient predicting suicidal ideation by entrapment

“*c’*” is the coefficient predicting suicidal ideation from panic-dissociation independent of entrapment

**Table 6:** Total, direct and indirect effects and effects sizes for indirect effects of panic-dissociation on suicidal ideation mediated by entrapment

	Raw effect	SE	lower	upper	*p*	CS	SE	κ^2^	SE	Z Score
Indirect effect	0.072	0.015	0.044	0.105	<0.001	0.211	0.0451	0.190	0.039	4.573
Direct effect	0.006	0.026	−0.045	0.056	0.821					
Total effect	0.077	0.024	0.031	0.124	0.001					

*SE* standard error, *lower* lower bound of 95% confidence interval, *upper* upper bound of 95% confidence interval, *CS* completely standardized effect size, *κ*^*2*^ Kappa-squared test, Z score by Sobel test

As indicated in Table 11, the reversed mediation analysis with entrapment as the independent variable and panic-dissociation as the mediator showed no indirect effect (Sobel test: *p* = 0.822) while both the direct effect and the total effect of entrapment on SI were significant. This confirms that entrapment fully mediates the relationship between panic-dissociation and SI, and this relationship is unidirectional.

#### Fear of dying

As indicated in Tables 7 and 8, the total effect of fear of dying on SI was significant at *p* = 0.041 with a standardized regression coefficient effect of 0.117. The direct effect of fear of dying on SI was not significant as the corresponding confidence interval includes zero. In contrast, the indirect effect of fear of dying on SI was significant (95% CI did not include 0 and Sobel test: z = 5.058, *p* < 0.001).

**Table 7:** Regression coefficients of entrapment and fear of dying predicting suicidal ideation

Predicted variable								
M(entrapment)					Y (suicidal ideation)			
Predictor variable		Coeff.	SE	P		Coeff.	SE	P
*X (Fear of dying)*	*a*	1.959	0.229	<0.001	*c’*	−0.082	0.061	0.177
*M (Entrapment)*		–	–	–	*b*	0.102	0.016	<0.001
*Constant*	*i*_*1*_	22.413	1.452	<0.001	*i*_*2*_	0.524	0.488	0.284
R^2^ = 0.270					R^2^ = 0.186			
F(1,198) = 73.161, *P* = <0.001					F(2,197) = 22.476, *P* < 0.001			

“*a*” is the regression coefficient predicting entrapment by fear of dying

“*b*” is the coefficient predicting suicidal ideation by entrapment

“*c’*” is the coefficient predicting suicidal ideation from fear of dying independent of entrapment

**Table 8:** Total, direct and indirect effects and effects sizes for indirect effects of fear of dying on suicidal ideation mediated by entrapment

	Raw effect	SE	lower	upper	*p*	CS	SE	κ^2^	SE	Z Score
Indirect effect	0.199	0.040	0.129	0.286	<0.001	0.247	0.049	0.222	0.042	5.058
Direct effect	−0.082	0.061	−0.202	0.038	0.178					
Total effect	0.117	0.057	0.005	0.229	0.041					

*SE* standard error, *lower* lower bound of 95% confidence interval, *upper* upper bound of 95% confidence interval, *CS* completely standardized effect size, *κ*^*2*^ Kappa-squared test, Z score by Sobel test

As indicated in Table 11, the reversed mediation analysis with entrapment as the independent variable and fear of dying as the mediator showed no significant indirect effect (Sobel test: *p* = 0.185). Both the direct effect and the total effect of entrapment on SI were significant. This indicates that entrapment fully mediates the relationship between fear of dying and SI, and fear of dying is not a mediator of the relationship between entrapment and SI.

#### Emotional pain

As indicated in Tables 9 and 10, the total effect of emotional pain on SI was significant at *p* < 0.001 with a standardized regression coefficient effect of 0.250. In contrast to the other SCS constructs, the direct effect of emotional pain on SI was significant with a standardized regression coefficient effect of 0.141, *p =* 0.009. Nevertheless, the indirect effect was also significant (95% CI did not include 0 and Sobel test: z = 2.774, *p =* 0.006), indicating that entrapment is a *partial* mediator of the relationship between emotional pain and SI.

**Table 9:** Regression coefficients of entrapment and emotional pain predicting suicidal ideation

Predicted variable								
M (entrapment)					Y (suicidal ideation)			
Predictor variable		Coeff.	SE	P		Coeff.	SE	P
*X (Emotional pain)*	*a*	1.986	0.141	<0.001	*c’*	0.141	0.054	0.009
*M (Entrapment)*		–	–	–	*b*	0.055	0.019	0.005
*Constant*	*i*_*1*_	13.358	1.525	<0.001	*i*_*2*_	0.279	0.486	0.566
R^2^ = 0.502					R^2^ = 0.206			
F(1,198) = 199.332, *P* = <0.001					F(2,197) = 25.549, *P* < 0.001			

“*a*” is the regression coefficient predicting entrapment by emotional pain

“*b*” is the coefficient predicting suicidal ideation by entrapment

“*c’*” is the coefficient predicting suicidal ideation from emotional pain independent of entrapment

**Table 10:** Total, direct and indirect effects and effects sizes for indirect effects of emotional pain on suicidal ideation mediated by entrapment

	Raw effect	SE	lower	upper	*p*	CS	SE	κ^2^	SE	Z Score
Indirect effect	0.108	0.042	0.027	0.193	0.006	0.181	0.070	0.140	0.052	2.774
Direct effect	0.141	0.054	0.035	0.248	0.009					
Total effect	0.250	0.039	0.173	0.326	<0.001					

*SE* standard error, *lower* lower bound of 95% confidence interval, *upper* upper bound of 95% confidence interval, *CS* completely standardized effect size, *κ*^*2*^ Kappa-squared test, Z score by Sobel test

Partial mediation by entrapment was further confirmed by the reversed mediation analyses with entrapment entered as the independent variable and emotional pain as the mediator. As shown in Table 11, while the total effect of entrapment on SI was significant (*p* < 0.001), both the direct effect (*p* = 0.005) and the indirect effect (Sobel test: *p =* 0.010) were also significant, which suggests that the mediation is not unidirectional. Thus, the mediating relationships of emotional pain and entrapment with SI appear to be reciprocal.

**Table 11:** Regression models for mediation of entrapment effects on SI by SCS components (reversed analysis)

Mediating variable	Total effect of Entrapment on SI: coefficient effect; (T score)	Direct effect of Entrapment on SI: coefficient effect; (T score)	Indirect effect of Entrapment on SI by Normal theory test: coefficient effect; (Z score)
Ruminative flooding	0.090; (6.553)^***^	0.082; (4.393)^***^	0.009; (0.683)
Panic-dissociation	0.090; (6.550)^***^	0.088; (5.509)^***^	0.002; (0.225)
Fear of dying	0.090; (6.550)^***^	0.102; (6.314)^***^	−0.011; (−1.327)
Emotional pain	0.090; (6.553)^***^	0.055; (2.836)^**^	0.036; (2.573)^*^

*SI* severity of suicidal ideation, *SCS* suicide crisis syndrome

^*^p < 0.05, ^**^p < 0.01, ^***^p < 0.001

### Supplementary analyses

In supplementary analyses, no differences were found in SI between patients admitted with SA and patients admitted with SI, nor were there substantive differences in the strength of mediation effects.

When age and depression severity were added as covariates to the mediation models the mediation effects of entrapment remained significant for each SCI subscale except emotional pain. (See Additional file 1.) Both entrapment and emotional pain, however, were independent predictors of SI (in separate models controlling for depression), and partially mediated the relation between depression severity and SI severity. (See Additional file 2.)

## Discussion

The primary aim of the present study was to test the hypothesis that entrapment mediates the relationships between other constructs of the SCS (ruminative flooding, panic-dissociation, fear of dying, and emotional pain) and SI. To our knowledge, this is the first study to show that entrapment plays a central role in the relationship between the symptoms of the SCS and SI in acutely suicidal individuals. Further, ruminative flooding, panic-dissociation, and fear of dying did not significantly mediate the effect of entrapment on SI, indicating that entrapment fully mediated the relationships between each of these constructs and SI. However, entrapment was only a partial mediator of the relationship between emotional pain and SI, and emotional pain was a significant partial mediator of the effect of entrapment on SI. This suggests both common and independent effects of entrapment and emotional pain on SI.

These results are consistent with our previous work, which has documented the prominence of entrapment in several populations of acutely suicidal patients both in the psychiatric emergency department and among psychiatrically hospitalized patients [11, 13]. Our data also support the central role of entrapment in the SCS, as assessed by the Suicide Crisis Inventory [12].

Ruminations have been previously linked to SI and suicidal behavior [25, 48, 49] although prior research has also pointed to an indirect rather than direct effect. Smith, Alloy [50] demonstrated that hopelessness partially mediated the relationship between ruminations and SI, and fully mediated the association between ruminations and duration of suicidality. Notably, Teismann and Forkmann [51] reported no direct relationship between ruminations and SI in two outpatient populations, as the link between the two was fully mediated by entrapment. Our results also show that the relationship between the ruminative flooding subscale of the SCI and SI is fully mediated by entrapment. This finding complements that by Teismann and Forkmann [51], and suggests that as ruminations during a suicidal crisis become uncontrollable and overwhelming, the perception of being trapped may precipitate SI.

Similarly, although numerous studies have shown that the experience of somatic symptoms, [11, 13, 27, 28, 52] and fear of dying [29–34], particularly in the context of anxiety and panic, predict SI or suicidal behavior, our current study indicates that in the acute suicidal state there are no direct linkages between either somatic symptoms or fear of dying and SI. Our results indicate instead that the somatic symptoms of panic, dissociation, and fear of dying are indirectly linked with SI via a pathway of entrapment. Thus, as with ruminative flooding, experiencing a fear of dying and dissociative symptoms may increase or simply be correlated with the intensity of stressful experiences that result in a sense of entrapment, which in turn might trigger SI.

Our results show that the relationship between emotional pain and SI was only partially mediated by entrapment, indicating a direct link between emotional pain and SI. Furthermore, the finding that the association between entrapment and SI was partially mediated by emotional pain suggests that these two constructs have both a direct and mutually mediatory relationship to SI. It may be that both of these constructs substantially entail the other. Namely, emotional pain may reliably produce the escape motivation that is necessary for entrapment, and the perception of entrapment may necessarily induce emotional pain. This understanding of the findings is consistent with Baumeister’s Escape Theory of Suicide [53] which conceptualizes suicide as an escape from otherwise seemingly inescapable painful self-states. Likewise, Shneidman [35] defines emotional pain or mental pain as a compilation of negative emotions (e.g., anxiety, helplessness, and despair) that reflects frustration with one’s inability to fulfill basic psychological needs. In Orbach and Mikulincer’s formulation, emotional pain includes loss of self-esteem, as well as feelings of failure, abandonment, emotional flooding, and emptiness and, notably, irreversibility [36–39]. Thus, emotional pain as assessed in studies using the Orbach Mikulincer Mental Pain scale, appears to be substantially convergent with the entrapment subscale of the SCI. The emotional pain subscale of the SCI, on the other hand, comprises only items directly descriptive of an emotional experience described in terms of pain. Past research has indicated that emotional pain mediates the relationship between perfectionism and suicidality [54], between hopelessness and SI [55], and between non-suicidal self-injury and suicide risk [56]. Shneidman [35], and later Orbach [36] further suggested that unbearable psychological pain may mediate the relationships between other relevant psychological factors and suicide. Similarly, in Klonsky & May’s recent Three Step Theory of suicide, the combination of pain and hopelessness drives the development of suicidal ideation; our findings for entrapment, which may be understood as a product of emotional pain and hopelessness, are thus also broadly consistent with the three-step theory [57]. Orbach’s studies and the strong correlation we found between entrapment and emotional pain suggest that entrapment is an emotionally painful experience. At the same time, however, the aversive experience of emotional pain may drive an urgency for escape which itself is requisite for the experience of entrapment. Our finding in supplementary analyses that controlling for severity of depression in mediation models of entrapment and emotional pain reduced mediation effects to non-significance points to the variance common to all three constructs. Nonetheless, entrapment and emotional pain each show incremental prediction of SI with respect to depression severity, highlighting the pertinence of each of these constructs.

Thus, taken altogether, our data indicate that in the SCS, entrapment acts as a conduit between most of the associated symptoms and SI. This finding is highly consistent with recent theoretical models of suicide including the Cry of Pain Model [14, 58] and the IMV Model of suicide [15], as well as Baumeister’s Escape Theory of Suicide [53] and Gilbert’s phenomenon of ‘arrested flight’ [16] and Galynker’s Narrative Crisis model [6]. Williams and Pollock [14] posit that suicide results from a feeling of defeat in response to humiliation or rejection, which in turn leads to a perception of entrapment. When the latter is combined with a failure to find alternative ways to solve a problem, i.e. cognitive rigidity [59], a suicidal person may see no exit out of his or her perceived entrapment other than suicide. One interpretation of the mediating role of entrapment is that perceptions of entrapment may constitute a final common pathway to SI. Therefore, therapeutic interventions aimed at reducing the perception of entrapment and creating actionable alternatives may eventually reduce suicide risk. In addition, clinical evaluation of entrapment, as illustrated in our previous reports [11, 13] by explicitly asking whether a patient felt “trapped,” or that “there was no way out,” or that he or she “had no good options,” would be essential for a suicide risk assessment. Further, our data suggest that in addition to explicitly asking whether patients feel trapped in their current life situation, relationship, or within themselves, it is important to assess whether they have experienced fear of dying, felt unusual sensations in their bodies or pressure inside their heads, and lost the ability to control and stop their ruminative thoughts, as such experiences were robustly correlated with entrapment and may provide a path of approach to assessment of suicidal thoughts that the patient might not otherwise disclose.

Based on our current results, and our findings from the complementary study of prediction of suicidal behavior [12], it can be hypothesized that while either unbearable emotional pain or entrapment may mediate the relationship between other symptoms of the SCS and SI, it is entrapment that may be the final mediator of the relationship of multiple psychological factors and suicidal action. A mediation analysis of prospective suicidal behavior using a larger sample size may provide support for this testable hypothesis. As the SCS is proposed to be a syndrome, the strong inter-correlations among the SCI subscales are certainly supportive of the SCS concept. Nonetheless, further study is needed to determine whether such a syndrome is more predictive of STB than simple severity of entrapment.

The results of this study need to be considered in the context of its limitations. This study represents a novel analysis of our previously published scale-validation data and thus all conclusions are tempered by the need for replication. Further, the study examines the relationship between the SCS constructs and SI rather than suicidal behavior or actual suicide, making the results less relevant to the urgent clinical task of suicide prevention. However, both active and passive SI have been shown to predict death by suicide marking it as a significant modifiable element of suicide risk [40, 41]; thus, understanding the mediation effects of the relationships of psychiatric symptoms in acutely suicidal individuals with SI can improve our understanding of the suicidal process in general. Second, the cross-sectional design of the study limits any causal interpretation of our findings. Third, the current study utilized only a self-report measure of SI, and the data may be confounded if some participants were not forthcoming in revealing their suicidality. Finally, the study was conducted at a single site, and study findings need to be replicated at other locations.

## Conclusion

Within its limitations, the current study highlights the central links between SI and entrapment and emotional pain in high-risk patients. The results suggest that psychiatric symptoms of a suicide crisis, such as ruminative flooding, panic-dissociation, and fear of dying may result in SI when they are experienced as painful states lacking routes for escape. The study further suggests that for individuals in a state of suicide crisis, the psychological processes tying entrapment and emotional pain to SI are highly similar, and generally consistent with models conceptualizing suicide as an escape behavior. Further research is warranted to establish the respective mediatory roles of entrapment and emotional pain with regard to future suicidal behavior.

## Additional files

Additional file 1: Mediation models controlling for age and depression. Results of mediation analyses controlling for age and depression. (DOC 63 kb)

Additional file 2: Mediation models demonstrating mutual partial mediation of entrapment and emotional pain by depression. Results of mediation analyses demonstrating mutual partial mediation of entrapment and emotional pain by depression. (DOC 57 kb)

## Abbreviations

BSS: Beck Scale for Suicide Ideation
IMV: Integrated Motivational-Volitional Model
MSBI: Mount Sinai Beth Israel
SCI: Suicide Crisis Inventory
SCS: Suicide Crisis Syndrome
SI: Suicidal Ideation
STB: Suicidal Thoughts and Behaviors
