# Supplementary material for: Entrapment as a mediator of suicide crises
Source: BMC Psychiatry. 2018 Jan 8;18:4. doi: 10.1186/s12888-018-1587-0 (PMC5759206; doi:10.1186/s12888-018-1587-0)
Supplement: Supplementary file 1 — Mediation models controlling for age and depression. Results of mediation analyses controlling for age and depression. (DOC 63 kb) [file 12888_2018_1587_MOESM1_ESM.doc]

Supplementary material: Mediation models controlling for age and depression

MEDIATION MODEL 1 - Ruminative Flooding
**************************************************************************
    Y = @1BSSst1 = SI
    X = @1STSrum = Ruminative Flooding
    M = @1STStrp = Entrapment

Statistical Controls:
BDIwoSI = Depression;  age


Sample size
        200

**************************************************************************
Outcome: @1STStrp

Model Summary
          R       R-sq        MSE          F        df1        df2          p
      .7066      .4993   106.2392    65.1620     3.0000   196.0000      .0000

Model
              coeff         se          t          p       LLCI       ULCI
constant     9.5142     2.4578     3.8710      .0001     4.6671    14.3613
@1STSrum      .9479      .1057     8.9658      .0000      .7394     1.1564
BDIwoSI       .2974      .0694     4.2843      .0000      .1605      .4344
age           .0212      .0552      .3843      .7011     -.0876      .1300

**************************************************************************
Outcome: @1BSSst1

Model Summary
          R       R-sq        MSE          F        df1        df2          p
      .5601      .3137     6.6858    22.2802     4.0000   195.0000      .0000

Model
              coeff         se          t          p       LLCI       ULCI
constant     -.6769      .6397    -1.0582      .2913    -1.9385      .5847
@1STStrp      .0487      .0179     2.7163      .0072      .0133      .0840
@1STSrum     -.0275      .0315     -.8720      .3843     -.0896      .0346
BDIwoSI       .1080      .0182     5.9301      .0000      .0721      .1439
age           .0127      .0138      .9201      .3587     -.0146      .0400

************************** TOTAL EFFECT MODEL ****************************
Outcome: @1BSSst1

Model Summary
          R       R-sq        MSE          F        df1        df2          p
      .5364      .2877     6.9034    26.3888     3.0000   196.0000      .0000

Model
              coeff         se          t          p       LLCI       ULCI
constant     -.2138      .6265     -.3413      .7332    -1.4494     1.0217
@1STSrum      .0187      .0269      .6929      .4892     -.0345      .0718
BDIwoSI       .1225      .0177     6.9211      .0000      .0876      .1574
age           .0138      .0141      .9792      .3287     -.0140      .0415

***************** TOTAL, DIRECT, AND INDIRECT EFFECTS ********************

Total effect of X on Y
     Effect         SE          t          p       LLCI       ULCI
      .0187      .0269      .6929      .4892     -.0345      .0718

Direct effect of X on Y
     Effect         SE          t          p       LLCI       ULCI
     -.0275      .0315     -.8720      .3843     -.0896      .0346

Indirect effect of X on Y
             Effect    Boot SE   BootLLCI   BootULCI
@1STStrp      .0461      .0195      .0079      .0856

Partially standardized indirect effect of X on Y
             Effect    Boot SE   BootLLCI   BootULCI
@1STStrp      .0176      .0074      .0029      .0321

Completely standardized indirect effect of X on Y
             Effect    Boot SE   BootLLCI   BootULCI
@1STStrp      .1221      .0511      .0212      .2247

Ratio of indirect to total effect of X on Y
             Effect    Boot SE   BootLLCI   BootULCI
@1STStrp     2.4708   132.2382      .2900  2033.8359

Ratio of indirect to direct effect of X on Y
             Effect    Boot SE   BootLLCI   BootULCI
@1STStrp    -1.6799    75.9305  -238.5943      .4796

Normal theory tests for indirect effect
     Effect         se          Z          p
      .0461      .0178     2.5849      .0097

******************** ANALYSIS NOTES AND WARNINGS *************************

Number of bootstrap samples for bias corrected bootstrap confidence intervals:
     5000

Level of confidence for all confidence intervals in output:
    95.00

MEDIATION MODEL 2 - Panic/Dissociation
**************************************************************************
    Y = @1BSSst1 = SI
    X = @1STSdis = Panic/Dissociation
    M = @1STStrp = Entrapment

Statistical Controls:
BDIwoSI = Depression;  age


Sample size
        200

**************************************************************************
Outcome: @1STStrp

Model Summary
          R       R-sq        MSE          F        df1        df2          p
      .6420      .4122   124.7297    45.8167     3.0000   196.0000      .0000

Model
              coeff         se          t          p       LLCI       ULCI
constant    15.8380     2.5061     6.3198      .0000    10.8956    20.7803
@1STSdis      .5905      .0941     6.2779      .0000      .4050      .7760
BDIwoSI       .4839      .0678     7.1322      .0000      .3501      .6177
age          -.0376      .0606     -.6214      .5351     -.1571      .0818

**************************************************************************
Outcome: @1BSSst1

Model Summary
          R       R-sq        MSE          F        df1        df2          p
      .5585      .3119     6.7029    22.0988     4.0000   195.0000      .0000

Model
              coeff         se          t          p       LLCI       ULCI
constant     -.7859      .6374    -1.2330      .2191    -2.0430      .4712
@1STStrp      .0437      .0166     2.6393      .0090      .0110      .0764
@1STSdis     -.0122      .0239     -.5103      .6104     -.0593      .0349
BDIwoSI       .1045      .0177     5.9226      .0000      .0697      .1394
age           .0141      .0141      .9999      .3186     -.0137      .0418

************************** TOTAL EFFECT MODEL ****************************
Outcome: @1BSSst1

Model Summary
          R       R-sq        MSE          F        df1        df2          p
      .5360      .2873     6.9070    26.3412     3.0000   196.0000      .0000

Model
              coeff         se          t          p       LLCI       ULCI
constant     -.0937      .5897     -.1590      .8739    -1.2568     1.0693
@1STSdis      .0136      .0221      .6150      .5393     -.0300      .0573
BDIwoSI       .1257      .0160     7.8726      .0000      .0942      .1572
age           .0124      .0143      .8706      .3850     -.0157      .0405

***************** TOTAL, DIRECT, AND INDIRECT EFFECTS ********************

Total effect of X on Y
     Effect         SE          t          p       LLCI       ULCI
      .0136      .0221      .6150      .5393     -.0300      .0573

Direct effect of X on Y
     Effect         SE          t          p       LLCI       ULCI
     -.0122      .0239     -.5103      .6104     -.0593      .0349

Indirect effect of X on Y
             Effect    Boot SE   BootLLCI   BootULCI
@1STStrp      .0258      .0118      .0052      .0522

Partially standardized indirect effect of X on Y
             Effect    Boot SE   BootLLCI   BootULCI
@1STStrp      .0098      .0044      .0018      .0191

Completely standardized indirect effect of X on Y
             Effect    Boot SE   BootLLCI   BootULCI
@1STStrp      .0832      .0376      .0169      .1633

Ratio of indirect to total effect of X on Y
             Effect    Boot SE   BootLLCI   BootULCI
@1STStrp     1.8958    49.5168      .2209   436.7248

Ratio of indirect to direct effect of X on Y
             Effect    Boot SE   BootLLCI   BootULCI
@1STStrp    -2.1163   160.6585 -10357.409     -.6410

Normal theory tests for indirect effect
     Effect         se          Z          p
      .0258      .0107     2.4072      .0161

******************** ANALYSIS NOTES AND WARNINGS *************************

Number of bootstrap samples for bias corrected bootstrap confidence intervals:
     5000

Level of confidence for all confidence intervals in output:
    95.00

NOTE: Some cases were deleted due to missing data.  The number of such cases was:
  1


MEDIATION MODEL 3 - Fear of Dying
**************************************************************************
    Y = @1BSSst1 = SI
    X = @1STSfrd = Fear of Dying
    M = @1STStrp = Entrapment

Statistical Controls:
BDIwoSI = Depression;  age

Sample size
        200

**************************************************************************
Outcome: @1STStrp

Model Summary
          R       R-sq        MSE          F        df1        df2          p
      .6551      .4291   121.1410    49.1095     3.0000   196.0000      .0000

Model
              coeff         se          t          p       LLCI       ULCI
constant    14.5237     2.4914     5.8295      .0000     9.6102    19.4371
@1STSfrd     1.4712      .2160     6.8107      .0000     1.0452     1.8972
BDIwoSI       .4887      .0662     7.3816      .0000      .3582      .6193
age          -.0253      .0593     -.4257      .6708     -.1423      .0918

**************************************************************************
Outcome: @1BSSst1

Model Summary
          R       R-sq        MSE          F        df1        df2          p
      .5687      .3234     6.5910    23.3023     4.0000   195.0000      .0000

Model
              coeff         se          t          p       LLCI       ULCI
constant     -.8000      .6295    -1.2708      .2053    -2.0415      .4415
@1STStrp      .0540      .0167     3.2430      .0014      .0212      .0869
@1STSfrd     -.1060      .0560    -1.8914      .0601     -.2165      .0045
BDIwoSI       .1049      .0175     6.0074      .0000      .0704      .1393
age           .0161      .0138     1.1591      .2478     -.0113      .0434

************************** TOTAL EFFECT MODEL ****************************
Outcome: @1BSSst1

Model Summary
          R       R-sq        MSE          F        df1        df2          p
      .5356      .2869     6.9110    26.2877     3.0000   196.0000      .0000

Model
              coeff         se          t          p       LLCI       ULCI
constant     -.0152      .5951     -.0256      .9796    -1.1888     1.1583
@1STSfrd     -.0265      .0516     -.5134      .6083     -.1282      .0753
BDIwoSI       .1313      .0158     8.3020      .0000      .1001      .1625
age           .0147      .0142     1.0362      .3014     -.0133      .0426

***************** TOTAL, DIRECT, AND INDIRECT EFFECTS ********************

Total effect of X on Y
     Effect         SE          t          p       LLCI       ULCI
     -.0265      .0516     -.5134      .6083     -.1282      .0753

Direct effect of X on Y
     Effect         SE          t          p       LLCI       ULCI
     -.1060      .0560    -1.8914      .0601     -.2165      .0045

Indirect effect of X on Y
             Effect    Boot SE   BootLLCI   BootULCI
@1STStrp      .0795      .0307      .0272      .1511

Partially standardized indirect effect of X on Y
             Effect    Boot SE   BootLLCI   BootULCI
@1STStrp      .0303      .0114      .0100      .0551

Completely standardized indirect effect of X on Y
             Effect    Boot SE   BootLLCI   BootULCI
@1STStrp      .1100      .0414      .0384      .2010

Ratio of indirect to total effect of X on Y
             Effect    Boot SE   BootLLCI   BootULCI
@1STStrp    -3.0012   150.8430 -2576.9972     -.6097

Ratio of indirect to direct effect of X on Y
             Effect    Boot SE   BootLLCI   BootULCI
@1STStrp     -.7501    21.1922    -5.1785     -.1633

Normal theory tests for indirect effect
     Effect         se          Z          p
      .0795      .0274     2.9026      .0037

******************** ANALYSIS NOTES AND WARNINGS *************************

Number of bootstrap samples for bias corrected bootstrap confidence intervals:
     5000

Level of confidence for all confidence intervals in output:
    95.00

NOTE: Some cases were deleted due to missing data.  The number of such cases was:
  1


MEDIATION MODEL 4 – Emotional Pain **************************************************************************
    Y = @1BSSst1 = SI
    X = @1STSepn = Emotional Pain
    M = @1STStrp = Entrapment

Statistical Controls:
BDIwoSI = Depression;  age

Sample size
        200

**************************************************************************
Outcome: @1STStrp

Model Summary
          R       R-sq        MSE          F        df1        df2          p
      .7359      .5416    97.2731    77.1903     3.0000   196.0000      .0000

Model
              coeff         se          t          p       LLCI       ULCI
constant    10.4812     2.2991     4.5589      .0000     5.9471    15.0153
@1STSepn     1.6410      .1595    10.2889      .0000     1.3264     1.9555
BDIwoSI       .2717      .0661     4.1086      .0001      .1413      .4021
age          -.0023      .0529     -.0433      .9655     -.1065      .1019

**************************************************************************
Outcome: @1BSSst1

Model Summary
          R       R-sq        MSE          F        df1        df2          p
      .5635      .3176     6.6477    22.6870     4.0000   195.0000      .0000

Model
              coeff         se          t          p       LLCI       ULCI
constant     -.7831      .6321    -1.2389      .2169    -2.0297      .4635
@1STStrp      .0251      .0187     1.3430      .1808     -.0117      .0619
@1STSepn      .0710      .0517     1.3716      .1717     -.0311      .1730
BDIwoSI       .0981      .0180     5.4462      .0000      .0626      .1336
age           .0121      .0138      .8773      .3814     -.0151      .0394

************************** TOTAL EFFECT MODEL ****************************
Outcome: @1BSSst1

Model Summary
          R       R-sq        MSE          F        df1        df2          p
      .5579      .3113     6.6750    29.5270     3.0000   196.0000      .0000

Model
              coeff         se          t          p       LLCI       ULCI
constant     -.5203      .6023     -.8639      .3887    -1.7080      .6675
@1STSepn      .1121      .0418     2.6838      .0079      .0297      .1945
BDIwoSI       .1049      .0173     6.0576      .0000      .0708      .1391
age           .0121      .0138      .8714      .3846     -.0152      .0394

***************** TOTAL, DIRECT, AND INDIRECT EFFECTS ********************

Total effect of X on Y
     Effect         SE          t          p       LLCI       ULCI
      .1121      .0418     2.6838      .0079      .0297      .1945

Direct effect of X on Y
     Effect         SE          t          p       LLCI       ULCI
      .0710      .0517     1.3716      .1717     -.0311      .1730

Indirect effect of X on Y
             Effect    Boot SE   BootLLCI   BootULCI
@1STStrp      .0412      .0366     -.0289      .1144

Partially standardized indirect effect of X on Y
             Effect    Boot SE   BootLLCI   BootULCI
@1STStrp      .0157      .0139     -.0112      .0431

Completely standardized indirect effect of X on Y
             Effect    Boot SE   BootLLCI   BootULCI
@1STStrp      .0691      .0606     -.0491      .1874

Ratio of indirect to total effect of X on Y
             Effect    Boot SE   BootLLCI   BootULCI
@1STStrp      .3670     3.4121     -.2889     1.9198

Ratio of indirect to direct effect of X on Y
             Effect    Boot SE   BootLLCI   BootULCI
@1STStrp      .5798   161.7577    -2.3198    39.6808

Normal theory tests for indirect effect
     Effect         se          Z          p
      .0412      .0310     1.3256      .1850

******************** ANALYSIS NOTES AND WARNINGS *************************

Number of bootstrap samples for bias corrected bootstrap confidence intervals:
     5000

Level of confidence for all confidence intervals in output:
    95.00

NOTE: Some cases were deleted due to missing data.  The number of such cases was:
  1
