# Supplementary material for: Entrapment as a mediator of suicide crises
Source: BMC Psychiatry. 2018 Jan 8;18:4. doi: 10.1186/s12888-018-1587-0 (PMC5759206; doi:10.1186/s12888-018-1587-0)
Supplement: Supplementary file 2 — Mediation models demonstrating mutual partial mediation of entrapment and emotional pain by depression. Results of mediation analyses demonstrating mutual partial mediation of entrapment and emotional pain by depression. (DOC 57 kb) [file 12888_2018_1587_MOESM2_ESM.doc]

Supplementary material: Mediation models demonstrating mutual partial mediation of entrapment and emotional pain by depression

MEDIATION MODEL 1 – Emotional Pain by Depression **************************************************************************
    Y = @1BSSst1 = SI
    X = @1STSepn = Emotional Pain
    M = BDIwoSI = Depression

Sample size
        200

**************************************************************************
Outcome: BDIwoSI

Model Summary
          R       R-sq        MSE          F        df1        df2          p
      .5249      .2755   113.8757    75.2803     1.0000   198.0000      .0000

Model
              coeff         se          t          p       LLCI       ULCI
constant    10.8588     1.5908     6.8258      .0000     7.7216    13.9959
@1STSepn     1.2730      .1467     8.6764      .0000      .9837     1.5624

**************************************************************************
Outcome: @1BSSst1

Model Summary
          R       R-sq        MSE          F        df1        df2          p
      .5555      .3086     6.6669    43.9646     2.0000   197.0000      .0000

Model
              coeff         se          t          p       LLCI       ULCI
constant     -.1511      .4278     -.3533      .7243     -.9948      .6926
BDIwoSI       .1067      .0172     6.2035      .0000      .0728      .1406
@1STSepn      .1138      .0417     2.7295      .0069      .0316      .1961

************************** TOTAL EFFECT MODEL ****************************
Outcome: @1BSSst1

Model Summary
          R       R-sq        MSE          F        df1        df2          p
      .4166      .1735     7.9290    41.5749     1.0000   198.0000      .0000

Model
              coeff         se          t          p       LLCI       ULCI
constant     1.0072      .4198     2.3994      .0174      .1794     1.8350
@1STSepn      .2496      .0387     6.4479      .0000      .1733      .3260

***************** TOTAL, DIRECT, AND INDIRECT EFFECTS ********************

Total effect of X on Y
     Effect         SE          t          p       LLCI       ULCI
      .2496      .0387     6.4479      .0000      .1733      .3260

Direct effect of X on Y
     Effect         SE          t          p       LLCI       ULCI
      .1138      .0417     2.7295      .0069      .0316      .1961

Indirect effect of X on Y
            Effect    Boot SE   BootLLCI   BootULCI
BDIwoSI      .1358      .0255      .0894      .1900

Partially standardized indirect effect of X on Y
            Effect    Boot SE   BootLLCI   BootULCI
BDIwoSI      .0440      .0082      .0289      .0612

Completely standardized indirect effect of X on Y
            Effect    Boot SE   BootLLCI   BootULCI
BDIwoSI      .2266      .0423      .1503      .3180

Ratio of indirect to total effect of X on Y
            Effect    Boot SE   BootLLCI   BootULCI
BDIwoSI      .5440      .1344      .3258      .8511

Ratio of indirect to direct effect of X on Y
            Effect    Boot SE   BootLLCI   BootULCI
BDIwoSI     1.1929    12.5900      .4718     5.4258

R-squared mediation effect size (R-sq_med)
            Effect    Boot SE   BootLLCI   BootULCI
BDIwoSI      .1474      .0343      .0884      .2201

Preacher and Kelley (2011) Kappa-squared
            Effect    Boot SE   BootLLCI   BootULCI
BDIwoSI      .2140      .0378      .1450      .2926

Normal theory tests for indirect effect
     Effect         se          Z          p
      .1358      .0270     5.0243      .0000

******************** ANALYSIS NOTES AND WARNINGS *************************

Number of bootstrap samples for bias corrected bootstrap confidence intervals:
     5000

Level of confidence for all confidence intervals in output:
    95.00

NOTE: Some cases were deleted due to missing data.  The number of such cases was:
  1


MEDIATION MODEL 2 – Emotional Pain by Depression **************************************************************************
    Y = @1BSSst1 = SI
    X = BDIwoSI = Depression
    M = @1STSepn = Emotional Pain

Sample size
        200

**************************************************************************
Outcome: @1STSepn

Model Summary
          R       R-sq        MSE          F        df1        df2          p
      .5249      .2755    19.3559    75.2803     1.0000   198.0000      .0000

Model
              coeff         se          t          p       LLCI       ULCI
constant     4.5660      .6528     6.9949      .0000     3.2787     5.8532
BDIwoSI       .2164      .0249     8.6764      .0000      .1672      .2656

**************************************************************************
Outcome: @1BSSst1

Model Summary
          R       R-sq        MSE          F        df1        df2          p
      .5555      .3086     6.6669    43.9646     2.0000   197.0000      .0000

Model
              coeff         se          t          p       LLCI       ULCI
constant     -.1511      .4278     -.3533      .7243     -.9948      .6926
@1STSepn      .1138      .0417     2.7295      .0069      .0316      .1961
BDIwoSI       .1067      .0172     6.2035      .0000      .0728      .1406

************************** TOTAL EFFECT MODEL ****************************
Outcome: @1BSSst1

Model Summary
          R       R-sq        MSE          F        df1        df2          p
      .5315      .2825     6.8840    77.9402     1.0000   198.0000      .0000

Model
              coeff         se          t          p       LLCI       ULCI
constant      .3687      .3893      .9470      .3448     -.3990     1.1363
BDIwoSI       .1313      .0149     8.8284      .0000      .1020      .1606

***************** TOTAL, DIRECT, AND INDIRECT EFFECTS ********************

Total effect of X on Y
     Effect         SE          t          p       LLCI       ULCI
      .1313      .0149     8.8284      .0000      .1020      .1606

Direct effect of X on Y
     Effect         SE          t          p       LLCI       ULCI
      .1067      .0172     6.2035      .0000      .0728      .1406

Indirect effect of X on Y
             Effect    Boot SE   BootLLCI   BootULCI
@1STSepn      .0246      .0103      .0064      .0467

Partially standardized indirect effect of X on Y
             Effect    Boot SE   BootLLCI   BootULCI
@1STSepn      .0080      .0033      .0021      .0151

Completely standardized indirect effect of X on Y
             Effect    Boot SE   BootLLCI   BootULCI
@1STSepn      .0997      .0411      .0275      .1890

Ratio of indirect to total effect of X on Y
             Effect    Boot SE   BootLLCI   BootULCI
@1STSepn      .1876      .0853      .0477      .3813

Ratio of indirect to direct effect of X on Y
             Effect    Boot SE   BootLLCI   BootULCI
@1STSepn      .2309      .1453      .0501      .6163

R-squared mediation effect size (R-sq_med)
             Effect    Boot SE   BootLLCI   BootULCI
@1STSepn      .1474      .0343      .0884      .2201

Preacher and Kelley (2011) Kappa-squared
             Effect    Boot SE   BootLLCI   BootULCI
@1STSepn      .1011      .0382      .0293      .1789

Normal theory tests for indirect effect
     Effect         se          Z          p
      .0246      .0095     2.5881      .0097

******************** ANALYSIS NOTES AND WARNINGS *************************

Number of bootstrap samples for bias corrected bootstrap confidence intervals:
     5000

Level of confidence for all confidence intervals in output:
    95.00

NOTE: Some cases were deleted due to missing data.  The number of such cases was:
  1


MEDIATION MODEL 3 – Entrapment by Depression
**************************************************************************
    Y = @1BSSst1 = SI
    X = @1STStrp = Entrapment
    M = BDIwoSI = Depression

Sample size
        200

**************************************************************************
Outcome: BDIwoSI

Model Summary
          R       R-sq        MSE          F        df1        df2          p
      .5418      .2936   111.0332    82.2765     1.0000   198.0000      .0000

Model
              coeff         se          t          p       LLCI       ULCI
constant     7.8651     1.8284     4.3017      .0000     4.2595    11.4706
@1STStrp      .4687      .0517     9.0706      .0000      .3668      .5706

**************************************************************************
Outcome: @1BSSst1

Model Summary
          R       R-sq        MSE          F        df1        df2          p
      .5549      .3080     6.6731    43.8313     2.0000   197.0000      .0000

Model
              coeff         se          t          p       LLCI       ULCI
constant     -.3582      .4687     -.7643      .4456    -1.2826      .5661
BDIwoSI       .1059      .0174     6.0769      .0000      .0715      .1402
@1STStrp      .0406      .0151     2.6942      .0077      .0109      .0703

************************** TOTAL EFFECT MODEL ****************************
Outcome: @1BSSst1

Model Summary
          R       R-sq        MSE          F        df1        df2          p
      .4222      .1782     7.8840    42.9416     1.0000   198.0000      .0000

Model
              coeff         se          t          p       LLCI       ULCI
constant      .4745      .4872      .9739      .3313     -.4863     1.4353
@1STStrp      .0902      .0138     6.5530      .0000      .0631      .1174

***************** TOTAL, DIRECT, AND INDIRECT EFFECTS ********************

Total effect of X on Y
     Effect         SE          t          p       LLCI       ULCI
      .0902      .0138     6.5530      .0000      .0631      .1174

Direct effect of X on Y
     Effect         SE          t          p       LLCI       ULCI
      .0406      .0151     2.6942      .0077      .0109      .0703

Indirect effect of X on Y
            Effect    Boot SE   BootLLCI   BootULCI
BDIwoSI      .0496      .0102      .0311      .0709

Partially standardized indirect effect of X on Y
            Effect    Boot SE   BootLLCI   BootULCI
BDIwoSI      .0161      .0032      .0101      .0226

Completely standardized indirect effect of X on Y
            Effect    Boot SE   BootLLCI   BootULCI
BDIwoSI      .2322      .0477      .1445      .3287

Ratio of indirect to total effect of X on Y
            Effect    Boot SE   BootLLCI   BootULCI
BDIwoSI      .5500      .1410      .3257      .8708

Ratio of indirect to direct effect of X on Y
            Effect    Boot SE   BootLLCI   BootULCI
BDIwoSI     1.2221   143.0739      .4639     6.1158

R-squared mediation effect size (R-sq_med)
            Effect    Boot SE   BootLLCI   BootULCI
BDIwoSI      .1527      .0332      .0947      .2249

Preacher and Kelley (2011) Kappa-squared
            Effect    Boot SE   BootLLCI   BootULCI
BDIwoSI      .2169      .0408      .1418      .3008

Normal theory tests for indirect effect
     Effect         se          Z          p
      .0496      .0099     5.0276      .0000

******************** ANALYSIS NOTES AND WARNINGS *************************

Number of bootstrap samples for bias corrected bootstrap confidence intervals:
     5000

Level of confidence for all confidence intervals in output:
    95.00

NOTE: Some cases were deleted due to missing data.  The number of such cases was:
  1


MEDIATION MODEL 4 – Depression by Entrapment
**************************************************************************
    Y = @1BSSst1 = SI
    X = BDIwoSI = Depression
    M = @1STStrp = Entrapment

Sample size
        200

**************************************************************************
Outcome: @1STStrp

Model Summary
          R       R-sq        MSE          F        df1        df2          p
      .5418      .2936   148.3933    82.2765     1.0000   198.0000      .0000

Model
              coeff         se          t          p       LLCI       ULCI
constant    17.9024     1.8074     9.9051      .0000    14.3382    21.4666
BDIwoSI       .6264      .0691     9.0706      .0000      .4902      .7625

**************************************************************************
Outcome: @1BSSst1

Model Summary
          R       R-sq        MSE          F        df1        df2          p
      .5549      .3080     6.6731    43.8313     2.0000   197.0000      .0000

Model
              coeff         se          t          p       LLCI       ULCI
constant     -.3582      .4687     -.7643      .4456    -1.2826      .5661
@1STStrp      .0406      .0151     2.6942      .0077      .0109      .0703
BDIwoSI       .1059      .0174     6.0769      .0000      .0715      .1402

************************** TOTAL EFFECT MODEL ****************************
Outcome: @1BSSst1

Model Summary
          R       R-sq        MSE          F        df1        df2          p
      .5315      .2825     6.8840    77.9402     1.0000   198.0000      .0000

Model
              coeff         se          t          p       LLCI       ULCI
constant      .3687      .3893      .9470      .3448     -.3990     1.1363
BDIwoSI       .1313      .0149     8.8284      .0000      .1020      .1606

***************** TOTAL, DIRECT, AND INDIRECT EFFECTS ********************

Total effect of X on Y
     Effect         SE          t          p       LLCI       ULCI
      .1313      .0149     8.8284      .0000      .1020      .1606

Direct effect of X on Y
     Effect         SE          t          p       LLCI       ULCI
      .1059      .0174     6.0769      .0000      .0715      .1402

Indirect effect of X on Y
             Effect    Boot SE   BootLLCI   BootULCI
@1STStrp      .0254      .0100      .0067      .0456

Partially standardized indirect effect of X on Y
             Effect    Boot SE   BootLLCI   BootULCI
@1STStrp      .0082      .0032      .0021      .0147

Completely standardized indirect effect of X on Y
             Effect    Boot SE   BootLLCI   BootULCI
@1STStrp      .1029      .0401      .0267      .1846

Ratio of indirect to total effect of X on Y
             Effect    Boot SE   BootLLCI   BootULCI
@1STStrp      .1937      .0833      .0468      .3731

Ratio of indirect to direct effect of X on Y
             Effect    Boot SE   BootLLCI   BootULCI
@1STStrp      .2402      .1404      .0491      .5950

R-squared mediation effect size (R-sq_med)
             Effect    Boot SE   BootLLCI   BootULCI
@1STStrp      .1527      .0332      .0947      .2249

Preacher and Kelley (2011) Kappa-squared
             Effect    Boot SE   BootLLCI   BootULCI
@1STStrp      .1029      .0376      .0266      .1758

Normal theory tests for indirect effect
     Effect         se          Z          p
      .0254      .0099     2.5684      .0102

******************** ANALYSIS NOTES AND WARNINGS *************************

Number of bootstrap samples for bias corrected bootstrap confidence intervals:
     5000

Level of confidence for all confidence intervals in output:
    95.00

NOTE: Some cases were deleted due to missing data.  The number of such cases was:
  1
